# Systemic Lupus Erythematosus and Pregnancy: A Single-Center Observational Study of 69 Pregnancies

**DOI:** 10.1055/s-0038-1672136

**Published:** 2018-10

**Authors:** Estephania Pignaton Naseri, Fernanda Garanhani Surita, Anderson Borovac-Pinheiro, Marília Santos, Simone Appenzeller, Lilian Tereza Lavras Costallat

**Affiliations:** 1 Department of Internal Medicine, Universidade Estadual de Campinas, Campinas, São Paulo, Brazil; 2 Department of Obstetrics and Gynecology, Universidade Estadual de Campinas, Campinas, São Paulo, Brazil

**Keywords:** systemic lupus erythematosus, pregnancy, obstetric complications, perinatal death, lúpus eritematoso sistêmico, gravidez, complicações obstétricas, morte perinatal

## Abstract

**Objective** To evaluate the effects of pregnancy in systemic lupus erythematosus (SLE) patients.

**Methods** The present article is a retrospective cohort study. Data were collected from medical records of pregnant women with SLE from January 2002 to December 2012 at Universidade Estadual de Campinas, in the city of Campinas, state of São Paulo, Brazil. Systemic lupus erythematosus and disease activity were defined according to the American College of Rheumatology and the Systemic Lupus Erythematosus Disease Activity Index (SLEDAI) criteria respectively. The means, standard deviations (SDs), percentages and correlations were performed using the SAS software, version 9.4 (SAS Institute Inc., Cary, NC, US).

**Results** We obtained data from 69 pregnancies in 58 women. During pregnancy, a new flare was observed in 39.2% (*n* = 27). The manifestations were most common in patients with prior kidney disease, and mainly occurred during the third quarter and the puerperium. Renal activity occurred in 24.6% (*n* = 17), and serious activity, in 16% (*n* = 11). Of all deliveries, 75% (*n* = 48) were by cesarean section. Two maternal deaths occurred (3%). Preterm birth was the main complication in the newborns. The abortion rate was 8.7%. Severe SLEDAI during pregnancy was associated with prematurity (100%) and perinatal death (54%).

**Conclusion** The maternal-fetal outcome is worse in SLE when the women experience a flare during pregnancy. The best maternal-fetal outcomes occur when the disease is in remission for at least 6 months before the pregnancy.

## Abstract (pt)

**Resumo:**

**Objetivo** Avaliar os efeitos da gravidez em pacientes com lúpus eritematoso sistêmico (LES).

**Métodos** Estudo de coorte retrospectivo. Os dados foram coletados de prontuários de mulheres com LES que engravidaram de janeiro de 2002 a dezembro de 2012 na Universidade Estadual de Campinas, São Paulo, Brasil. Lúpus eritematoso sistêmico e atividade da doença foram definidos segundo o American College of Rheumatology e os critérios do Índice de Atividade da Doença de Lúpus Eritematoso (SLEDAI, na sigla em inglês), respectivamente. As médias, os desvios-padrão (DP), as porcentagens e as correlações foram realizados utilizando o software SAS, versão 9.4 (SAS Institute Inc., Cary, NC, US).

**Resultados** Obtivemos dados de 69 gestações em 58 mulheres. Durante a gravidez, a reatividade da doença foi observada em 39.2% (*n* = 27). As manifestações mais comuns foram em pacientes com doença renal prévia, e ocorreram principalmente no terceiro trimestre e no puerpério. Atividade renal ocorreu em 24,6% (*n* = 17), e atividade grave, em 16% (*n* = 11). De todos os partos, 75% (*n* = 48) foram por cesariana. Dois óbitos maternos ocorreram (3%). A prematuridade foi a principal complicação nos recém-nascidos. A taxa de aborto foi de 8,7%. O índice SLEDAI grave durante a gestação foi associado à prematuridade (100%) e à morte perinatal (54%).

**Conclusão** O resultado materno-fetal é pior no LES quando as mulheres sofrem crise de reativação durante a gravidez. Os melhores desfechos materno-fetais ocorrem quando a doença está em remissão por pelo menos 6 meses anteriores à gestação.

## Introduction

Systemic lupus erythematosus (SLE) is a chronic multisystem autoimmune disease that mainly affects women of childbearing age. However, their fertility, regardless of the severity of the disease, is preserved.^1^ ^2^

In patients with rheumatic autoimmune diseases, particularly SLE, pregnancy presents a challenge for the physicians. Pregnant women with lupus have a higher mortality risk and a greater risk of eclampsia, preeclampsia, preterm birth, and thromboembolic events, in addition to an increase in the disease activity itself. In pregnant SLE patients, complications associated with the disease are often difficult to distinguish from physiological changes or complications arising from the pregnancy itself.^2^ ^3^

The association of SLE and antiphospholipid antibodies and antiphospholipid antibody syndrome (APS) further increases the probability of miscarriage, restricted intrauterine growth, mortality, and perinatal complications.^4^ These antibodies are present in ∼ 25–50% of all SLE patients, but only a small proportion of patients with antiphospholipid antibodies develop APS.^4^ ^5^ The presence of the anticoagulant isolated antibody is more helpful for predicting the risk of a negative outcome in such cases.^5^ Three decades ago, female SLE patients would simply be discouraged from getting pregnant. However, several studies, particularly those evaluating the use of antimalarial drugs during pregnancy, have shown favorable maternal-fetal outcomes in cases of SLE. For successful outcomes, the participation of a multidisciplinary team involving rheumatologists, obstetricians, and pediatricians is required, although this is not feasible in developing countries.^1^ ^6^ ^7^

In the present study, we evaluated SLE patients whose prenatal care was administered by a team of experts in rheumatology and high-risk obstetrics of Hospital de Clínicas da Universidade Estadual de Campinas (HC/Unicamp), in the Portuguese acronym), in the city of Campinas, state of São Paulo, Brazil, to evaluate complications and outcomes in these patients.

## Methods

A retrospective cohort study was conducted at HC/Unicamp, which is a reference hospital center that covers an area with ∼ 4 million inhabitants. It performs ∼ 2,800 high-risk deliveries each year, and has an assistance protocol specifically designed for the care of pregnant women with SLE.

We included 58 women with SLE followed in the outpatient clinic of Rheumatology who became pregnant, totaling 69 pregnancies from January 2002 to December 2012. During the pregnancy and childbirth, these patients were followed in high-risk prenatal care at Centro de Atenção Integral à Saúde da Mulher (CAISM, in the Portuguese acronym), a center specialized in the care of female patients that is part of the HC/Unicamp.

All women were > 18 years old and met the classification criteria of the American College of Rheumatology (1997).^8^ We observed the newborns from birth until they were discharged from the hospital.

We have collected data from medical records using a data collection form, and disease activity was defined through the Systemic Lupus Disease Activity Index (SLEDAI. The calculation of the SLEDAI was made with data from 6 months prior to the beginning of the pregnancy and every time the patients showed changes in clinical and laboratory findings during pregnancy. Disease activity was considered mild to moderate when the SLEDAI was > 4 and severe when it was > 12.^9^

Data were incorporated in a Microsoft Excel (Microsoft Corporation, Redmond, WA, US) worksheet, and the statistical analysis was performed using the SAS software (SAS Institute Inc., Cary, NC, US), version 9.4, for Windows. Values of *p* < 0.05 were considered statistically significant.

The exploratory data analysis was developed by assessing frequency, percentages, averages, and standard deviation (SD). We used the Fisher exact test to establish correlations among variables.

The institutional review board assessed and approved the research protocol before data collection started (approval number 30310114.2.0000.5404).

## Results

We have obtained data from 69 pregnancies in 58 women: 16 (23%) had planned the pregnancy; however, all 100% of the pregnancies were desired. The average age was 33 years old (SD ± 7) at the time of delivery, and 76% (*n* = 53) of the sample was aged between 31 and 45 years. Of all patients, 65% (*n* = 45) received prenatal counseling. The average age was 23 years old (SD ± 5.8) at the time of SLE diagnosis, with an average duration of the disease of 4.45 years (SD ± 4.83). Systemic lupus erythematosus was diagnosed during pregnancy in 4 patients (5.8%). A prior abortion had occurred in 4% (*n* = 12), stillbirth, in 7.3% (*n* = 5), 34% (*n* = 24) were nulliparous, and ∼ 45% (*n* = 31) of the patients already had living children before the diagnosis. Table 1 shows SLE manifestations and drugs used before pregnancy. Upon the diagnosis of pregnancy, 20% of all patients ceased using immunosuppressant drugs, either by themselves or as per the suggestion of the diagnosing physician.

**Table 1:** SLE Manifestations and drugs used before pregnancy

	n (%)
Activity signs	
Antinuclear antibody	68 (98.6)
Arthritis	56 (81.2)
Immunologic change	50 (72.4)
Anti-antibody DNA	34 (42.2)
Photosensitivity	48 (69.6)
Hematologic change	37 (53.6)
Malar rash	35 (50.7)
Serositis	18 (26.0)
Renal involvement	18 (26.0)
Discoid lupus	9 (13.0)
Neurological involvement	8 (11.5)
Oral ulcers	6 (8.7)
**Drugs used**	63 (91.3)
Prednisone ≤ 20 mg/day	49 (71.0)
Hydroxychloroquine	23 (33.3)
Azathioprine	15 (21.7)
Chloroquine diphosphate	7 (10.1)
Prednisone ≥ 0.5 mg/Kg/day	6 (8.7)
Cyclophosphamide	3 (4.2)
Biological (abatacept)	1 (1.5)
Mycophenolate	1 (1.5)

Previous comorbidities, such as hypertension and diabetes, were present in 16 of 58 - 23% of all women; 12 (17.4%) women presented with associated APS, and 18 (26.1%) presented with 1 or more antiphospholipid antibodies with no clinical diagnosis of APS.

During pregnancy, a new flare (disease activity) was observed in 27 (39.2%) women, particularly during the 2nd and 3rd trimesters. Renal activity occurred in 24.6% (*n* = 17), and serious activity, in 16% (*n* = 11) of the women. Renal involvement is related with flare presentation (66%), with worsened renal function found in 41% of the cases after the flare. The activity of SLE during pregnancy and the fetal outcomes are shown in Table 2.

**Table 2:** Systemic lupus erythematosus activity during pregnancy and perinatal outcomes

	N (%)
Disease reactivation during pregnancy	27 (39.2)
1st quarter	3 (4.4)
2st quarter	8 (11.6)
3st quarter	16 (23.2)
Disease reactivation during the puerperium	7 (10.1)
Arthritis	9 (16.3)
Cutaneous	16 (29.0)
Hematological	14 (25.4)
Autoimmune hemolytic anemia	3 (5.4)
Thrombocytopenia	4 (7.2)
Leukopenia	7 (12.7)
Serositis	1 (1.8)
Renal Injury	14 (25.4)
Central nervous system vasculitis	1 (1.8)
Anti-antibody DNA	7 (10.1)
Complement consumption	27 (39.2)
Systemic Lupus Erythematosus Disease Activity Index*	
Activity	19 (27.9)
Severe activity	11 (16.2)
No activity	38 (55.9)
Fetal death	11 (15.9)
Abortion	5 (7.3)
up to 6	2 (4.6)
≥ 7	42 (95.5)
Preterm birth***	37 (59.4)
< 28 weeks	7 (10.9)
28–34 weeks	13 (20.3)
34– < 37 weeks	17 (26.6)
Intensive care unit admission****	7 (11.7)
Weight at birth****	
Small for gestational age	11 (18.3)
Adequate for gestational age	49 (81.7)
Large for gestational age	0 (0.0)
Birth defects	3 (4.3)

Notes: Missing: * 1, **25, ***5, ****9.

Of all deliveries, 75% (*n* = 48) were by cesarean section. Regarding pregnancy-associated complications, gestational diabetes was diagnosed in 2.9% (*n* = 2), pre-eclampsia in 10% (*n* = 7), 2 women (2.9%) had sepsis, and there were no cases of eclampsia or HELLP syndrome (hemolysis, elevated liver enzyme levels, and low platelet levels). Two (3%) maternal deaths occurred (Table 3).

**Table 3:** Perinatal outcomes according to pregestational and gestational activity of Systemic Lupus Erythematosus

	PREGESTATIONAL SLEDAI				GESTATIONAL SLEDAI			
	No activity	Activity	Severe activity	*p*-value	No activity	Activity	Severe activity	*p*-value
	n (%)	n (%)	n (%)		n (%)	n (%)	n (%)	
Mode of delivery*				1.00				0.37
C-section	35 (74.5)	9 (75.0)	4 (80.0)		28 (80.0)	13 (72.2)	6 (60.0)	
Vaginal	12 (25.5)	3 (25.0)	1 (20.0)		7 (20.0)	5 (27.8)	4 (40.0)	
Prematurity*				0.05				0.01
No	23 (48.9)	3 (25.0)	0 (0.0)		18 (51.4)	8 (44.4)	0 (0.0)	
Yes	24 (51.1)	9 (75.0)	5 (100.0)		17 (48.6)	10 (55.6)	10 (100.0)	
Apgar 5**				0.70				0.43
Up to 6	5 (14.7)	0 (0.0)	0 (0.0)		5 (16.7)	0 (0.0)	0 (0.0)	
≥7	29 (85.3)	7 (100.0)	5 (100.0)		25 (83.3)	10 (100.0)	4 (100.0)	
Perinatal death				0.07				< 0.01
No	44 (88.0)	1 (84.6)	3 (50.0)		38 (100.0)	15 (78.9)	5 (45.5)	
Yes	6 (12.0)	2 (15.4)	3 (50.0)		0 (0.0)	4 (21.1)	6 (54.5)	

Abbreviations: C-section, cesarean section; SLEDAI, Systemic Lupus Erythematosus Disease Activity Index.

Notes: missing: *5 **25.

After the delivery, almost all women (98%) received contraceptive guidelines and 62% immediately started a new contraceptive method, while 35% (20) opted for tubal ligation.

Birth defects were observed in 3 newborns, 1 of them with tetralogy of Fallot. In this case, the mother had lupus nephritis, and was being treated with intravenous cyclophosphamide. The other observed malformations were megaureter (*n* = 1) and hydronephrosis (*n* = 1). These two cases occurred in women without disease activity for six months prior to the pregnancy. Only one case of neonatal SLE was described, which presented with skin and hematological changes, without cardiac alterations.

## Discussion

The present study aimed to evaluate the effects of SLE previously and during pregnancy. The data showed that cutaneous and hematologic activities were the most common presentations previously and during pregnancy, and that almost all women were using prescription drugs during the conception period.

In our sample, 27 (39.2%) of the patients experienced a flare during pregnancy, an expected percentage according to previous studies (13–65%).^10^ In a previous study conducted at the same center and involving 76 pregnancies, flares occurred in 85% of the cases.^11^ We attribute this improvement to increased use of hydroxychloroquine by pregnant women in recent years, and to the lower renal involvement before the pregnancy presented by the patients in the study (26% in the present study versus 66% in the previous study). In patients with renal involvement prior to the pregnancy, 66% presented with a flare, and subsequent worsening of renal function was observed in 41% of the cases. These data are in line with previous studies showing that patients with SLE with and without prior nephritis presented a risk of flare of 54.2% versus 25% (*p* = 0.04), and exclusively renal flare in 45.7% versus 6.6% (*p* = 0.0001).^12^ ^13^ The majority of the disease activity observed in these patients ranged from mild to moderate, with new cutaneous-articular changes. The episodes of disease activity were mainly observed in the second and third trimesters.

Chen et al^14^ described 83 pregnancies of patients with SLE that were divided into 3 groups: patients in remission for more than 6 months, patients with disease activity in the previous 6 months, and patients who were diagnosed with SLE during pregnancy. In this study, the SLEDAI calculated six months prior to the pregnancy period was significantly associated with flare and fetal loss.^14^ In the present study, the SLEDAI evaluated at the moment the flare occurred had a significant association with perinatal death (*p* < 0.01); 50% of perinatal deaths occurred in those with severe disease activity, as shown by the SLEDAI, while 21% of the fetal deaths occurred in those with mild to moderate disease activity, and no fetal deaths occurred in patients without flare.

Systemic lupus erythematosus was diagnosed during pregnancy in four patients with severe disease activity and with severe maternal or maternal-fetal outcome. Fetal death occurred in three of them, and two patients died.

One woman died during the 12th week of pregnancy; this patient had had a previous miscarriage. This woman presented with severe thrombocytopenia and serositis. The other maternal death occurred during the 33rd week of pregnancy in a women with a high SLEDAI < 6 months prior to the pregnancy and an active lupus nephritis with worsened renal function during the pregnancy. Both died due to pulmonary sepsis shock secondary to a pulse of methylprednisolone. The main reason for maternal death in women with SLE found in the literature is pulmonary infection involved with sepsis.^15^ ^16^

The maternal death rate in our study is in line with the literature (3%).^15^ The main cause of maternal death is sepsis (40%), followed by disease activity (30%), and secondary causes include pulmonary embolism, cardiomyopathy, and kidney failure, which were less frequent.^15^ ^16^

Diabetes and hypertension were found as severe complications related with pregnant women with SLE . Nevertheless, although the cohort previously used corticosteroids, diabetes diagnosed during pregnancy was only observed in two cases, with no newborns large for gestational age. Preeclampsia was found in 7 (10%) women, and no difference was observed among women with flare and without flare during pregnancy. According to previous studies, the risk of preeclampsia in SLE patients ranges from 10 to 35%, which is similar to what was observed in the present study, although the risk of preeclampsia is particularly high in patients who have chronic kidney disease.^13^ ^17^

As expected, premature birth was the main complication observed in the newborns. Of all newborns, 37 (59%) were born prematurely, with extreme premature birth (< 28 weeks) in 7 of 69 (10%) of the cases. In previous studies, ∼ 40% of all newborns were born prematurely.^15^ High disease activity was the main predictor of preterm birth in women with SLE.^18^

Neonatal lupus was diagnosed only in one case in which the mother did not experience a flare during pregnancy, but had Anti-Ro and Anti-La antibodies. The fetus developed properly during the gestation, and was born at term, with an appearance, pulse, grimace, activity, and respiration (APGAR) score of 9 and 10 at 5 and 10 minutes of life respectively, showing only cutaneous manifestations and lymphopenia, without any cardiac change. Two studies show neonatal cutaneous involvement present in around 37% of all cases of neonatal lupus.^19^ ^20^ The prevalence of neonatal hematological changes is uncertain.^20^

## Conclusion

The present study assessed pregnancy in women with SLE and evaluated previous manifestations and perinatal outcomes. However, it has some limitations: data collection had no control, and we had some data loss because it was a retrospective study. Nevertheless, the study shows the improvement in the pattern of the outcome in pregnant women with SLE, especially after the introduction of hydroxychloroquine, when compared with data already existing in the literature. Pregnancy in women with SLE is considered of high risk, and that risk is even higher in unplanned pregnancies with high disease activity and when the disease is diagnosed during pregnancy. On these occasions, even with expert multidisciplinary follow-up, there is a great risk of premature birth and perinatal death.
